# Radiofrequency ablation for early breast cancer: from local technique to system-based surgical therapy

**DOI:** 10.3389/fonc.2026.1893430

**Published:** 2026-08-25

**Authors:** Shinichiro Kashiwagi

**Affiliations:** Department of Breast Surgical Oncology, Osaka Metropolitan University Graduate School of Medicine, Osaka, Japan

**Keywords:** breast cancer, image-guided therapy, local control, minimally invasive therapy, radiofrequency ablation, rescue surgery, thermal ablation, ultrasound guidance

## Abstract

Radiofrequency ablation (RFA) is a minimally invasive local treatment option for selected patients with early breast cancer, with potential advantages in cosmetic preservation and reduced physical burden. Breast RFA nevertheless requires organ-specific adaptation because abundant adipose tissue, superficial tumor location, and proximity to the skin and chest wall influence electrical impedance, heat conduction, ablation geometry, and safety. A meta-analysis of 17 studies involving 399 patients and 401 lesions smaller than 2 cm reported 99% technical success, 96% complete ablation, a mean ablation time of 15.8 minutes, and a pooled complication rate of 2%; however, most studies were small, and 65.7% of patients underwent post-ablation surgical excision. More recently, the multicenter, single-arm phase 3 RAFAELO study treated 370 patients with Tis–T1 tumors measuring 1.5 cm or less and reported 98.6% 5-year ipsilateral breast tumor recurrence-free survival among 353 patients completing follow-up, with two ipsilateral recurrences. These results are encouraging but remain conditioned by strict selection, mandatory radiotherapy, protocolized surveillance, and the absence of a concurrent randomized surgical control. This review therefore frames breast RFA as a system-based local therapy rather than an isolated technical maneuver. It details causes and recognizable patterns of insufficient ablation, proposes a minimum framework for reproducible technical and reporting standardization, summarizes quantitative efficacy and safety outcomes, and emphasizes post-ablation imaging, biopsy when indicated, and predefined rescue strategies. Breast RFA should not be regarded as a universal replacement for breast-conserving surgery; its safe use requires multidisciplinary selection, reproducible image-guided execution, objective procedural documentation, structured surveillance, and prospective comparative validation.

## Introduction

Breast-conserving surgery (BCS) combined with radiotherapy has been established as a standard local treatment for early breast cancer, providing excellent oncological outcomes with acceptable cosmetic results. Nevertheless, surgical excision inevitably involves tissue removal and skin incision, which may affect cosmetic outcomes and patient experience. In recent years, there has been growing interest in minimally invasive local therapies aimed at reducing treatment-related physical burden while maintaining oncological safety.

Radiofrequency ablation (RFA) is a thermal ablation technique that induces coagulative necrosis through Joule heating generated by high-frequency alternating current (1). RFA has been widely adopted in the treatment of hepatic tumors and other solid malignancies (2); however, direct extrapolation of these experiences to breast cancer is inappropriate. The breast is characterized by abundant adipose tissue, superficial tumor location, and proximity to the skin and chest wall, all of which significantly influence heat conduction, ablation geometry, and risk of thermal injury. These organ-specific characteristics necessitate dedicated technical considerations and procedural adaptations when applying RFA to breast cancer.

Early clinical studies have demonstrated the feasibility of breast RFA, particularly for small, well-defined tumors (3–6). Subsequent pathological and imaging-based evaluations further clarified the local effects and limitations of the technique (7–12). However, inconsistent ablation techniques, heterogeneous eligibility criteria, and variability in postprocedural assessment have limited broader adoption outside selected expert centers.

Although previous reviews have summarized the technical feasibility and oncological outcomes of breast RFA, less attention has been devoted to procedural standardization and its integration into multidisciplinary surgical pathways. This gap is clinically important because the primary challenge of breast RFA lies not only in achieving tumor necrosis, but also in reliably assessing treatment completeness and managing potential residual disease. Unlike surgical excision, RFA does not provide a resected specimen for pathological margin evaluation, underscoring the need for robust intra- and post-procedural evaluation strategies (8–12).

In this review, breast RFA is proposed to be better conceptualized as a system-based surgical therapy rather than as an isolated local technique. This concept does not imply that RFA is an accepted replacement for standard BCS; rather, it emphasizes that any safe implementation of breast RFA requires an integrated pathway incorporating patient selection, image-guided technique, intraprocedural endpoint assessment, post-ablation evaluation, and predefined options for additional treatment or surgical conversion when incomplete ablation is suspected.

The novelty of this review is its focus on the clinical system surrounding breast RFA. Specifically, the manuscript integrates technical execution, mechanisms of insufficient ablation, oncological assessment, society and regulatory positions, and a proposed clinical algorithm into a single framework intended to support safe, reproducible, and evidence-generating implementation.

## Literature search strategy and scope

A focused narrative literature search was performed to improve methodological transparency. PubMed/MEDLINE and Google Scholar were searched from database inception to July 20, 2026, using combinations of the following terms: “breast cancer,” “radiofrequency ablation,” “thermal ablation,” “non-surgical ablation,” “early breast cancer,” “complete ablation,” “insufficient ablation,” “breast density,” “impedance,” “heat conduction,” “technical standardization,” “reporting criteria,” “local control,” “ipsilateral breast tumor recurrence,” “complication,” “procedural outcome,” “imaging,” “MRI,” “microbubble,” “biopsy,” “rescue surgery,” and “guideline.” Reference lists of relevant reviews and clinical studies were manually screened. Priority was given to peer-reviewed clinical studies, prospective or multicenter experiences, systematic reviews or meta-analyses, consensus reporting documents, guideline publications, and reports published from 2023 onward. Because the aim was a conceptual and clinically oriented narrative review rather than a formal systematic review, study selection was not performed according to PRISMA, and no formal quantitative synthesis or risk-of-bias assessment was undertaken.

## Technical standardization of breast radiofrequency ablation

### Fundamental principles of electrode placement and energy delivery

**Figure 1:**
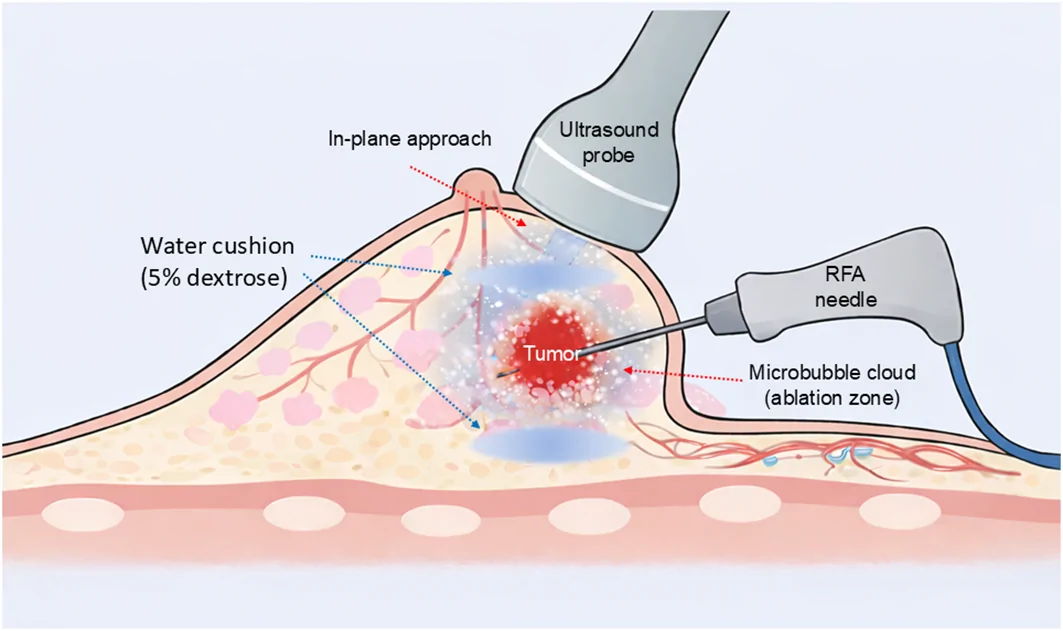
Ultrasound-guided radiofrequency ablation of early breast cancer. Schematic illustration of ultrasound-guided radiofrequency ablation (RFA) for early breast cancer. The electrode is inserted into the tumor using an in-plane approach, allowing continuous visualization of the needle under real-time ultrasound guidance. During ablation, microbubble distribution serves as a surrogate marker of the ablation zone. A water cushion created with 5% dextrose solution is used to reduce thermal injury to surrounding tissues. Continuous ultrasound monitoring is essential for procedural safety and reproducibility.

Accurate electrode placement is a central determinant of effective breast RFA. In contrast to surgical excision, for which margins are evaluated pathologically, RFA success depends heavily on the geometry achieved during the procedure. The electrode should be placed near the three-dimensional tumor center under real-time ultrasound guidance, preferably with an in-plane approach that permits continuous visualization of the shaft and tip. Before energy delivery, central placement should be documented in at least two orthogonal planes, with explicit measurement of the shortest distances to the skin, pectoral fascia, nipple-areolar complex, and tumor boundaries. Failure to confirm both the tip and the active electrode segment can produce eccentric heating even when a single ultrasound plane appears satisfactory (3, 4, 7–15) (**Figure 1**).

Energy delivery should be tailored to tumor size, breast composition, device characteristics, and safety constraints. During ablation, impedance and temperature trajectories provide complementary information regarding tissue heating, dehydration, and coagulative necrosis (1). A sudden impedance increase (“impedance break”) is an important cue, but it should not be interpreted in isolation because premature desiccation or high baseline impedance may terminate current delivery before the planned ablation volume is achieved. The pooled literature demonstrates that pathological completeness may remain imperfect despite technically completed procedures (16). In a 2026 series of 50 treated breasts, the mean peak temperature was 81.0 ± 8.0°C, the mean time to impedance break was 446 ± 139 seconds, and fat-rich breasts showed higher impedance, lower peak temperatures, and shorter ablation times, suggesting a tissue-composition-dependent risk of a narrow or insufficient ablation zone (17).

### Microbubble distribution as an intraprocedural surrogate endpoint

**Figure 2:**
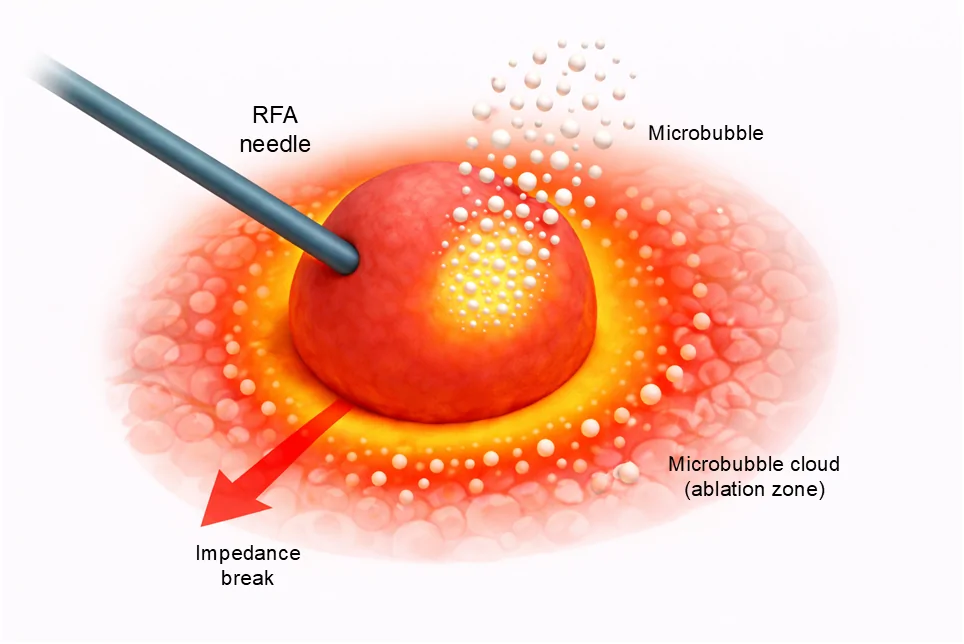
Microbubble-based endpoint during radiofrequency ablation Schematic illustration of the ablation endpoint during breast RFA. Adequate ablation is indicated by circumferential distribution of microbubbles around the tumor, reflecting thermal coverage of both the target lesion and the planned safety margin. The appearance of a circumferential microbubble cloud, together with impedance changes during energy delivery, is used as an intraprocedural surrogate indicator for determining completion of ablation.

In breast RFA, visualization of microbubble formation plays a central role in assessing ablation extent. Microbubbles generated during tissue heating appear as hyperechoic signals on ultrasound and serve as a real-time surrogate marker of the ablation zone. Uniform distribution of microbubbles encompassing the target lesion and surrounding safety margin has been used in clinical practice as an intraprocedural indicator of thermal coverage (**Figure 2**). Unlike post-procedural imaging alone, intraprocedural microbubble assessment enables immediate feedback and may allow operators to adjust needle position or energy delivery before terminating ablation.

Importantly, microbubble coverage should be interpreted as a procedural surrogate rather than a validated predictor of durable local control. The expanding echogenic cloud may itself obscure the posterior or peripheral tumor boundary, and apparent circumferential coverage in one plane can coexist with an untreated sector in another. Assessment should therefore include repeated sweeps in orthogonal planes, comparison with the pre-ablation tumor map, and documentation of the relationship between the cloud and the planned margin before visualization is lost. Microbubble appearance is influenced by breast composition, lesion depth, device and electrode design, energy-delivery pattern, and operator experience. It must be integrated with impedance and temperature data, post-ablation imaging, biopsy when indicated, and clinical follow-up rather than used as a standalone guarantee of complete ablation (10, 16–19).

### Thermal protection and safety-oriented implementation

Thermal injury to the skin, subcutaneous tissue, nipple-areolar complex, and chest wall is a major breast-specific concern. A protective fluid cushion created with 5% dextrose in water is commonly used to separate vulnerable structures from the anticipated heat field. Because 5% dextrose has substantially lower ionic conductivity than saline, it is preferred when hydrodissection is required around a radiofrequency circuit (20). The protective layer should be confirmed throughout the procedure because tissue deformation, fluid redistribution, or electrode movement can reduce separation. Continuous ultrasound monitoring, direct observation of the skin, and predefined stopping criteria for unexpected superficial bubble extension, pain, or device abnormalities are essential. Importantly, safety-driven reductions in power or early termination may prevent thermal injury but can also increase the risk of under-ablation; this trade-off should trigger enhanced post-ablation verification rather than an assumption of technical success.

### Technical pitfalls and patterns of insufficient ablation

**Figure 3:**
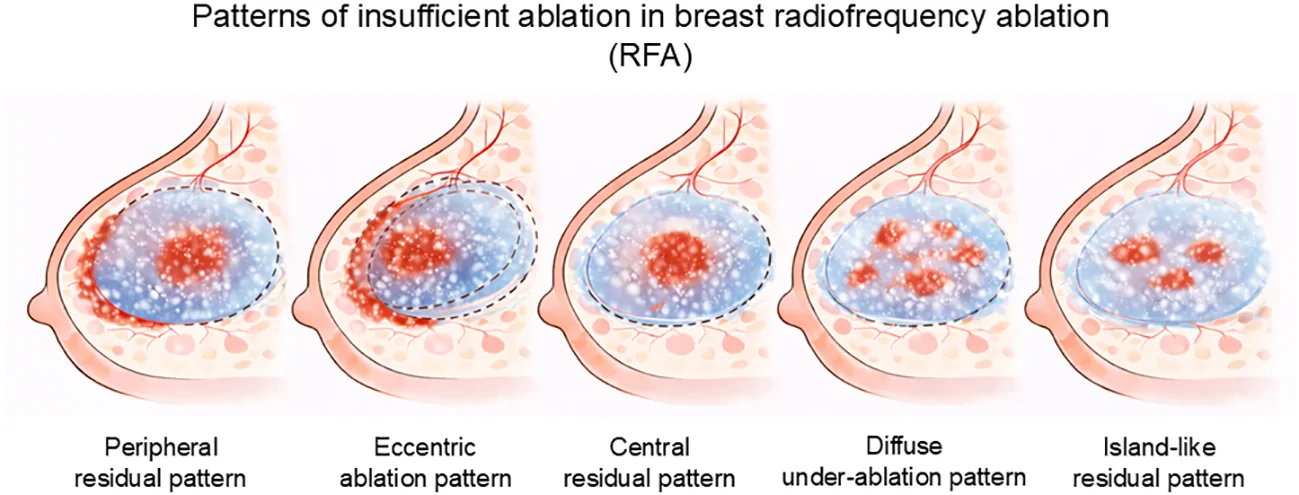
Patterns of insufficient ablation in breast radiofrequency ablation. Representative schematic illustrations of typical patterns of insufficient ablation observed after breast RFA. Five major patterns are shown: peripheral residual pattern, eccentric ablation pattern, central residual pattern, diffuse under-ablation pattern, and island-like residual pattern. These patterns reflect different mechanisms of inadequate thermal coverage related to needle positioning, energy delivery, heat-sink effects, tissue visibility, and heterogeneous heat conduction. Clinical implications and corrective actions are summarized in **Table 1** to avoid overcrowding the schematic figure.

Insufficient ablation can arise from interacting geometric, tissue-related, monitoring, and biological factors. Geometric failure includes off-center electrode placement, an inappropriate insertion angle or depth, lesion displacement during hydrodissection, and failure to visualize the complete active tip. Early ablate-and-resect studies documented residual viable tumor after technically completed procedures and identified probe position, tumor size, and the difficulty of monitoring a progressively echogenic target as plausible contributors (3, 4, 7–15). These mechanisms commonly produce peripheral or eccentric residual patterns and underscore the need for preplanned three-dimensional targeting rather than reliance on a single central ultrasound image (**Table 1**; **Figure 3**).

**Table 1:** Mechanisms, warning signs, and corrective strategies for insufficient ablation in breast radiofrequency ablation.

Pattern of insufficient ablation	Mechanism, warning signs, clinical implication, and corrective strategy
Peripheral residual pattern	Mechanism: inadequate circumferential thermal coverage, underestimation of the imaging-defined tumor extent, or an occult intraductal component beyond the visible mass. Warning signs: incomplete microbubble extension at one margin or a narrow planned margin near the skin or chest wall. Clinical implication: marginal viable disease. Corrective strategy: reassess the lesion and planned margin in orthogonal planes; perform additional ablation only when safe; and use targeted post-ablation imaging and biopsy for a suspicious margin.
Eccentric ablation pattern	Mechanism: off-center electrode placement, suboptimal angle or depth, lesion displacement, or failure to confirm the needle tip in two orthogonal planes. Warning signs: asymmetric microbubble expansion and unequal distances from the electrode to the tumor boundary. Clinical implication: asymmetric residual disease despite apparently adequate central heating. Corrective strategy: document tumor-centered placement before energy delivery, reposition the electrode when necessary, and confirm circumferential coverage in multiple planes.
Central residual pattern	Mechanism: inadequate energy or duration, premature impedance rise caused by tissue desiccation or charring, high impedance in fat-rich tissue, or local perfusion-related heat loss. Warning signs: early impedance break, lower-than-expected temperature, or a persistent central hypoechoic area. Clinical implication: viable tumor within the intended target zone. Corrective strategy: review the complete power-temperature-impedance curve, device settings, and tissue composition; consider controlled repeat ablation within safety limits; and verify with post-ablation imaging or biopsy.
Diffuse under-ablation pattern	Mechanism: globally insufficient energy delivery, poor electrode-tissue contact, incorrect device settings, patient movement, poor sonographic visibility, or premature termination because the echogenic cloud obscures the target. Warning signs: limited or patchy microbubble formation and absent expected impedance or temperature change. Clinical implication: global treatment failure. Corrective strategy: stop and re-establish visualization, verify the system and electrode position, repeat treatment only if safety can be maintained, and arrange early biopsy-based confirmation or definitive rescue therapy.
Island-like residual pattern	Mechanism: heterogeneous electrical and thermal conductivity, variable perfusion, irregular tumor geometry, or microscopic intraductal/multifocal foci beyond the apparent ablation zone. Warning signs: discontinuous microbubble coverage or discordance between ultrasound and contrast-enhanced imaging. Clinical implication: scattered viable foci may be missed by limited sampling or imaging alone. Corrective strategy: use multimodal assessment, target suspicious foci for biopsy, and maintain protocol-defined imaging surveillance with a low threshold for definitive local therapy.

Tissue composition creates a second source of variability. Breast adipose and fibroglandular tissues differ in electrical impedance and thermal conductivity, and local perfusion may dissipate heat. Fat-rich tissue can exhibit higher impedance and lower achieved temperature, whereas premature tissue desiccation and charring can produce an early impedance break that limits further energy deposition. The recent breast-density analysis by Futamura et al. provides direct clinical evidence that these parameters vary systematically with tissue composition (17). Central or diffuse under-ablation should therefore prompt review of the entire power-temperature-impedance trajectory rather than merely confirmation that an impedance break occurred.

A third pitfall is endpoint misclassification. The hyperechoic microbubble cloud is useful for immediate feedback, but it may obscure the original lesion and does not directly demonstrate cell death. Similarly, early post-ablation enhancement, edema, fibrosis, or fat necrosis can complicate MRI and ultrasound interpretation, while imaging-only follow-up may miss microscopic viable foci that would be detected in an ablate-and-resect design (8–10, 18, 21). Discordance among procedural findings, imaging, and pathology should therefore be treated as uncertainty requiring targeted biopsy or definitive local therapy, not as a negative test.

Finally, image-defined tumor size may underestimate an extensive intraductal component or discontinuous microscopic disease. Poorly circumscribed lesions, multifocality, limited ultrasound visibility, close proximity to the skin or chest wall, patient movement, pain, and device-specific restrictions can further constrain the attainable margin. In the meta-analysis of 401 lesions smaller than 2 cm, technical failure was uncommon but was attributed to probe-placement difficulty, poor ultrasound visibility, patient cooperation, pain, and device-related factors; the pooled complete-ablation rate was 96%, demonstrating that technical completion is not equivalent to pathological completeness (16). **Table 1** links five recognizable insufficiency patterns with warning signs and corrective strategies.

### Toward reproducible technical standardization

Reproducibility requires standardization of the entire treatment pathway, not only the ablation maneuver. At minimum, pre-procedural documentation should include biopsy-confirmed histology and biomarkers; tumor dimensions in three axes; concordant ultrasound and contrast-enhanced MRI assessment of unifocality and disease extent; distances to the skin, chest wall, and nipple-areolar complex; breast density or tissue composition; nodal evaluation; and multidisciplinary confirmation that standard surgical and non-surgical options have been discussed. Eligibility exclusions and reasons for protocol deviation should be recorded prospectively.

The procedural protocol should specify the device, generator, electrode type and active-tip length, anesthesia, patient position, ultrasound approach, hydrodissection solution and volume, target electrode coordinates, planned ablation margin, power-escalation algorithm, maximum power, treatment duration, temperature and impedance sampling, definition of impedance break, criteria for repeat ablation, and stopping rules. Tumor-centered placement should be documented in orthogonal planes before energy delivery. During treatment, operators should archive representative images or cine loops showing electrode position, microbubble evolution, and the final apparent coverage. A device-specific protocol is preferable to an unvalidated universal energy prescription; for example, one recent Japanese protocol initiated treatment at 5 W, increased to 10 W after 1 minute, and then escalated by 5–10 W per minute according to tissue response (17).

Post-procedural standardization should prespecify the timing and modality of imaging, definitions of technical success and technique efficacy, biopsy indications and sampling method, adverse-event grading, criteria for incomplete ablation, and triggers for repeat ablation or surgical conversion. Outcome reporting should distinguish immediate technical success, pathologically or radiologically verified complete ablation, local tumor progression at the treated site, ipsilateral breast tumor recurrence elsewhere, rescue-treatment rate, cosmetic outcome, patient-reported outcome, disease-free survival, and overall survival. These terms should follow established image-guided ablation reporting principles wherever applicable (19, 20).

Institutional quality assurance should include formal operator training, supervised initial cases, a procedural checklist, multidisciplinary review of uncertain cases, image archiving, and periodic audit of completeness, complications, and rescue interventions. Japan’s regulated implementation and multicenter experience demonstrate that credentialing and facility requirements can support dissemination, but cross-device and cross-institution reproducibility still requires prospective validation (21, 22). These proposed elements represent a minimum evidence-generating framework rather than a universally validated international protocol.

### Clinical implementation and oncological assessment

#### Patient selection and procedural eligibility

Appropriate patient selection is fundamental to successful clinical implementation of breast RFA. Favorable features generally include a small, unifocal, image-visible tumor with clearly defined margins, clinical node negativity, and an anatomical location that permits safe needle placement and thermal protection. Caution is warranted for tumors with extensive intraductal components, poorly delineated imaging margins, multifocal or multicentric disease, lesions immediately adjacent to the skin or chest wall without adequate protective separation, or situations in which reliable follow-up imaging or biopsy cannot be ensured. These criteria should not be interpreted as universally accepted indications, but as practical eligibility considerations derived from available clinical experience and implementation frameworks (5–12, 22, 23).

#### Proposed clinical algorithm

**Figure 4:**
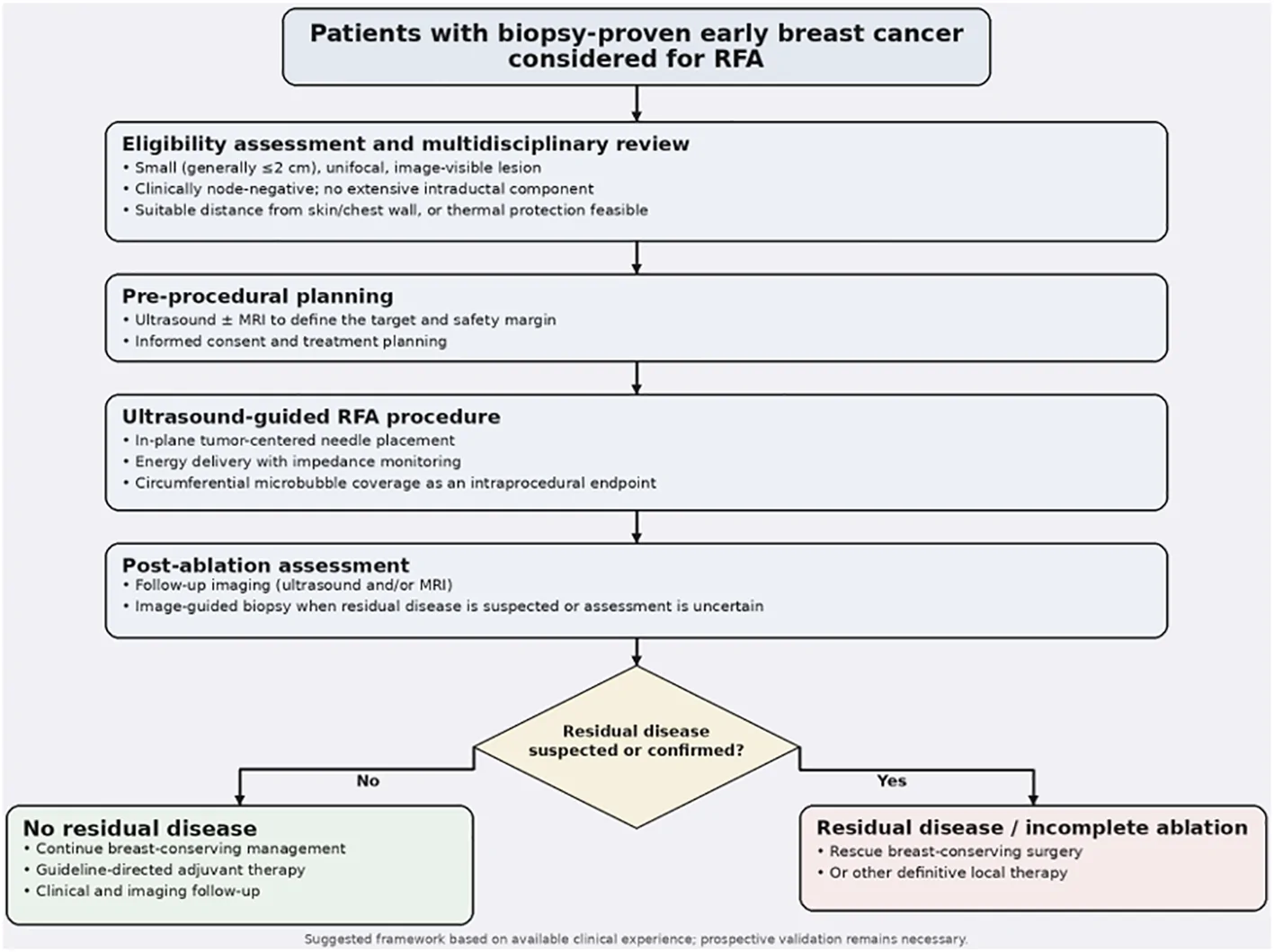
Proposed clinical algorithm for breast radiofrequency ablation in early breast cancer. Proposed clinical algorithm for integrating breast RFA into a structured management pathway. Patients with biopsy-proven early breast cancer considered for RFA undergo eligibility assessment and multidisciplinary review, followed by pre-procedural imaging and planning, ultrasound-guided RFA with intraprocedural assessment, and post-ablation imaging and biopsy when indicated. If residual disease or incomplete ablation is suspected or confirmed, rescue breast-conserving surgery or another definitive local therapy should be considered. If no residual disease is evident, breast-conserving management continues with guideline-directed adjuvant therapy and clinical-imaging follow-up. This framework is based on available clinical experience and requires prospective validation.

A proposed clinical algorithm is shown in **Figure 4**. The pathway begins with biopsy-proven early breast cancer considered for RFA and proceeds through eligibility assessment and multidisciplinary review, pre-procedural imaging and consent, ultrasound-guided RFA with intraprocedural endpoint assessment, and post-ablation surveillance. When residual disease is suspected or confirmed, rescue breast-conserving surgery or another definitive local therapy should be considered. When no residual disease is evident, breast-conserving management continues with guideline-directed adjuvant therapy and clinical-imaging follow-up. This algorithm is intended as a conceptual framework based on available clinical experience and requires prospective validation before broad adoption.

#### Post-ablation evaluation and oncological safety

Clinical implementation of breast RFA requires a structured framework for post-ablation assessment to ensure oncological safety. Because RFA does not yield a resected specimen for pathological margin evaluation, treatment success cannot be assumed solely based on intraprocedural findings. Instead, a combination of post-ablation imaging and tissue sampling when indicated plays a central role in confirming treatment completeness (10, 18). Imaging modalities such as ultrasound and magnetic resonance imaging are commonly used to evaluate the ablation zone, identify suspicious residual enhancement, and monitor post-treatment changes (18, 24, 25). However, imaging findings alone may be insufficient, particularly in the early post-ablation period when inflammatory changes, fibrosis, and fat necrosis can obscure residual viable tumor (10, 18). Image-guided biopsy therefore provides an additional layer of safety when residual disease is suspected or imaging assessment is uncertain (8, 9).

#### Rescue strategy as a safety component

A predefined rescue strategy is an important component of clinical breast RFA implementation, but it should not be framed as an obligatory intervention for all patients. When incomplete ablation or residual viable tumor is suspected or confirmed, surgical conversion to breast-conserving surgery is one management option, alongside other definitive local strategies selected according to tumor extent, patient preference, and multidisciplinary judgment (22, 23). The availability of rescue surgery transforms RFA from a potentially uncertain standalone intervention into a controlled treatment pathway with built-in safeguards. This concept reinforces that RFA is not intended to replace surgery indiscriminately, but to function within a system that prioritizes oncologic safety through timely escalation when necessary.

#### Integration into multidisciplinary breast cancer care

Breast RFA should be integrated into multidisciplinary breast cancer management rather than applied as an isolated intervention. Adjuvant radiotherapy and systemic therapies should be administered according to established clinical guidelines, independent of the local treatment modality. From this perspective, RFA functions as a local treatment component within a broader therapeutic strategy, rather than as a substitute for comprehensive cancer care (22, 23). Clinical experience has suggested that when implemented within a standardized framework—including precise intraprocedural execution, structured post-ablation assessment, and predefined rescue pathways—breast RFA may achieve favorable local control while preserving cosmetic outcomes in selected patients (22, 23). The conceptual differences between BCS and RFA are summarized in **Table 2**.

**Table 2:** Conceptual comparison between breast-conserving surgery and radiofrequency ablation for early breast cancer.

Aspect	Breast-conserving surgery (BCS)	Radiofrequency ablation (RFA)
Treatment concept	Surgical excision with pathological margin assessment	Image-guided thermal ablation without tissue excision
Invasiveness	Requires skin incision and tissue removal	Minimally invasive, usually without skin incision
Cosmetic outcome	May be affected by surgical scar and volume loss	Generally favorable cosmetic outcomes in appropriately selected patients
Local control	High and well-established local control	Promising in strictly selected cohorts; the single-arm RAFAELO study reported 98.6% 5-year ipsilateral breast tumor recurrence-free survival, but no randomized concurrent comparison with BCS is available (21).
Predictability	High predictability as a standard treatment	Dependent on eligibility, device and protocol adherence, operator experience, and reliable intra- and post-procedural assessment.
Treatment assessment	Pathological evaluation of resected specimen	Imaging-based evaluation with biopsy when indicated
Rescue strategy	Rarely required	Protocol-defined biopsy, repeat ablation, additional local therapy, or surgical conversion may be considered when completeness is uncertain or residual disease is confirmed.
Patient experience	Standard postoperative recovery	Potentially reduced physical burden and rapid recovery in selected patients
Educational perspective	Fundamental surgical technique	Advanced image-guided and minimally invasive surgical technique
Role in clinical practice	Established standard of care	Implemented under defined indications in Japan; internationally, it remains a complementary or investigational option rather than a universal standard alternative to BCS.
Future perspective	Stable position as standard treatment	Potential expansion through standardized protocols and reporting, prospective comparative validation, long-term follow-up, and evaluation of patient-reported outcomes and cost-effectiveness.

#### Current position of guideline and society frameworks

The current international position remains cautious. Contemporary early breast cancer guidelines continue to define surgery, radiotherapy, and systemic therapy as the core components of curative-intent management, and RFA is not established as a routine universal alternative to breast-conserving surgery (26–28). Japan has progressed further through protocolized multicenter evaluation, regulatory approval, public insurance coverage since 2023, defined eligibility criteria, facility requirements, and operator training (17, 21, 22). The definitive 2026 RAFAELO report strengthens the evidence base but does not eliminate the need for caution because it used a single-arm design, a historical control threshold, strict tumor-size selection, and mandatory radiotherapy. Thus, the Japanese experience is best interpreted as a model of controlled implementation rather than proof that breast RFA can be generalized to all early breast cancers.

#### Oncological outcomes and contemporary evidence

Quantitative outcomes must be interpreted according to study design and verification method (**Table 3**). In the prospective ablate-and-resect study by Ohtani et al., complete pathological ablation was achieved in 36 of 41 patients (87.8%), whereas MRI showed no residual enhancement in 25 of 26 evaluable patients (96.2%), illustrating that imaging and pathological estimates are not interchangeable (12). In the 10-institution retrospective study by Ito et al. (386 patients; median follow-up, 50 months), ipsilateral breast tumor recurrence occurred in 8 of 355 tumors measuring 2 cm or less (2.3%) and 3 of 30 tumors larger than 2 cm (10.0%); the corresponding 5-year recurrence-free rates were 97%, 94%, and 87% for tumors measuring 1.0 cm or less, 1.1–2.0 cm, and more than 2.0 cm, respectively (15).

**Table 3:** Quantitative outcomes from representative studies of radiofrequency ablation for early breast cancer.

Study and design	Population	Technical/procedural outcomes	Oncologic outcomes	Safety and interpretation
Ohtani et al. (2011) (12); prospective ablate-and-resect study	41 patients; tumors <2 cm	One RFA session completed in 40 patients; complete pathological ablation in 36/41 (87.8%); MRI showed no residual enhancement in 25/26 evaluable patients (96.2%)	Not designed to estimate long-term recurrence because delayed surgical resection was performed	One superficial skin burn; pathological verification was rigorous, but the study did not represent a definitive non-excisional pathway
Ito et al. (2018) (15); 10-institution retrospective study	386 patients; broad real-world tumor-size distribution	Technical details and assessment protocols varied among institutions	Median follow-up 50 months; ipsilateral breast tumor recurrence occurred in 8/355 tumors ≤2 cm (2.3%) and 3/30 tumors >2 cm (10.0%); 5-year recurrence-free rates were 97%, 94%, and 87% for tumors ≤1.0 cm, 1.1–2.0 cm, and >2.0 cm, respectively	Local pain in 9 patients, skin burns in 15, and nipple retraction in 7; retrospective design and protocol heterogeneity limit causal comparison
Xia et al. (2021) (16); systematic review and meta-analysis	17 studies; 399 patients and 401 lesions <2 cm	Pooled technical success 99%; complete ablation 96%; mean ablation time 15.8min; 65.7% underwent post-ablation surgical excision	No local recurrence among 232 patients with recurrence data at a median follow-up of 27.29 months	Pooled complication rate 2%; skin burns 2.02% (8/397); short follow-up, mixed verification methods, and publication bias require cautious interpretation
Takayama et al. (2023) (22); PO-RAFAELO implementation study	120 patients treated as of January 2023	Structured implementation under predefined facility and eligibility requirements	Long-term recurrence outcomes were not mature in this implementation report	No grade ≥3 early adverse events; supports feasibility of regulated dissemination but not comparative efficacy
Futamura et al. (2026) (17); single-institution retrospective technical study	49 patients; 50 treated breasts	Mean peak temperature 81.0 ± 8.0°C; mean time to impedance break 446 ± 139 s; initial and final impedance 208 ± 72.3 Ω and 161 ± 66.5 Ω	Not designed for long-term oncologic comparison	Fat-rich breasts had higher impedance, lower peak temperature, and shorter ablation time, identifying tissue composition as a potential cause of insufficient ablation
Kinoshita et al. (2026) (21); multicenter, single-arm phase 3 RAFAELO study	370 treated patients with Tis–T1 (≤1.5 cm), N0M0 disease; 353 completed 5-year follow-up	Protocol-defined RFA followed by radiotherapy (45–60 Gy)	5-year ipsilateral breast tumor recurrence-free survival 98.6% (95% CI 96.6–99.4%); two ipsilateral breast recurrences	One grade ≥3 skin ulceration among 370 patients; strong prospective evidence, but strict selection, mandatory radiotherapy, and a historical rather than concurrent control limit generalizability

The 2021 meta-analysis by Xia et al. included 17 studies, 399 patients, and 401 lesions smaller than 2 cm. Pooled technical success was 99%, complete ablation was 96%, mean ablation time was 15.8 minutes, and the pooled complication rate was 2%; skin burns occurred in 8 of 397 patients (2.02%) (16). No local recurrence was reported among 232 patients with recurrence data after a median follow-up of 27.29 months. Nevertheless, 261 of 397 patients (65.7%) underwent post-ablation surgical excision, and the pooled analysis combined pathological, imaging, and clinical definitions of completeness. These design features can inflate apparent efficacy and limit inference about a definitive non-excisional pathway.

The strongest long-term prospective evidence is now provided by the multicenter phase 3 RAFAELO study. Among 370 treated patients with Tis–T1, N0M0 tumors measuring 1.5 cm or less, 353 completed 5-year follow-up; the 5-year ipsilateral breast tumor recurrence-free survival rate was 98.6% (95% CI 96.6–99.4%), with two ipsilateral recurrences and one grade 3 or higher skin ulceration (21). All participants received 45–60 Gy of radiotherapy, and efficacy was evaluated against a prespecified historical threshold rather than a randomized concurrent breast-conserving surgery group. Accordingly, RAFAELO demonstrates that highly selected patients can achieve favorable 5-year local control within a tightly controlled Japanese pathway, but it does not establish equivalence across broader populations, devices, or follow-up strategies.

Procedural and safety data also vary by reporting method. The meta-analysis estimated a pooled complication rate of 2%, whereas individual multicenter and retrospective cohorts reported events such as pain, skin burns, nipple retraction, fat necrosis, or ulceration with different severity definitions and follow-up windows (15, 16, 21). **Table 3** therefore reports denominators, follow-up, verification strategy, and design limitations alongside headline efficacy figures to avoid false direct comparisons.

## Discussion

The available evidence now supports a more differentiated interpretation of breast RFA. Technical feasibility is high, pooled complete-ablation rates approach the mid-90% range, and the RAFAELO study reported 98.6% 5-year ipsilateral breast tumor recurrence-free survival in a strictly selected cohort (16, 21). These results move breast RFA beyond an experimental proof of concept. They do not, however, justify treating it as a universally interchangeable substitute for lumpectomy because outcomes are inseparable from tumor selection, radiotherapy, procedural quality control, verification strategy, and access to rescue treatment.

A key insight emerging from accumulated experience is that breast RFA differs fundamentally from surgical excision in how treatment completeness is assessed. Unlike BCS, which relies on pathological margin evaluation, RFA requires surrogate intra- and post-procedural indicators of efficacy. Intraprocedural assessment based on impedance changes and microbubble distribution provides real-time feedback, while post-ablation imaging and biopsy-based evaluation offer important safeguards for detecting residual disease (10, 18). However, these tools have limitations, and none can yet replace the evidentiary certainty provided by a resected specimen. This is the central reason that RFA should be discussed as a structured management pathway rather than as a simple technical substitute for lumpectomy.

The revised framework also clarifies the role of rescue surgery. Surgical conversion should be available when incomplete ablation or residual disease is suspected, but it should be regarded as one management option rather than an obligatory component in all cases. This distinction is important for patient counseling: RFA may reduce invasiveness for selected patients, but patients must understand that additional biopsy, imaging surveillance, or surgery may be required if treatment completeness cannot be confirmed.

Several clinically relevant questions remain unresolved. No adequately powered randomized trial has compared a definitive RFA pathway with contemporary breast-conserving surgery plus radiotherapy using harmonized endpoints. The optimal post-ablation MRI timing and biopsy strategy are not established; the learning curve and minimum case volume have not been quantified; device-specific protocols are not interchangeable; and the incremental cost of the electrode, anesthesia, imaging, biopsy, prolonged surveillance, and rescue treatment has not been weighed against cosmetic, recovery, and patient-preference benefits. Long-term disease-free survival, breast cancer-specific survival, patient-reported outcomes, and 10-year local control remain insufficiently characterized.

### Limitations of the existing evidence

The literature has substantial structural limitations. First, many studies are small, single-center, retrospective, or ablate-and-resect series in which surgery is performed soon after RFA. Such designs provide strong pathological verification but cannot estimate the effectiveness, surveillance burden, or delayed complications of a definitive non-excisional pathway. Conversely, non-surgical cohorts better reflect intended practice but are vulnerable to verification bias because only suspicious cases undergo biopsy or surgery. The pooled literature therefore combines fundamentally different clinical questions (12, 14–16, 29).

Second, definitions are heterogeneous. “Technical success,” “complete ablation,” “local recurrence,” and “complication” have been based variously on generator completion, ultrasound appearance, contrast-enhanced imaging, routine histology, viability stains, cytokeratin immunohistochemistry, core biopsy, or delayed clinical follow-up. Early thermal injury can preserve cellular morphology despite loss of viability, while limited sampling can miss small residual foci. This heterogeneity impedes pooled comparison and may explain differences between imaging and pathological estimates (8–10, 16, 18, 19).

Third, selection and treatment confounding are substantial. Most favorable cohorts restrict enrollment to small, unifocal, ultrasound-visible tumors with clearly defined MRI extent, and many patients have hormone receptor-positive disease. Radiotherapy and systemic therapy are commonly administered and contribute to local control, making the independent effect of RFA difficult to isolate. RAFAELO further used mandatory radiotherapy and a historical control rather than a concurrent surgical arm (21). Evidence is concentrated in expert Japanese and European centers, limiting generalizability to different breast compositions, devices, training systems, and health-care settings.

Fourth, reporting of learning curves, protocol deviations, failed procedures, rescue surgery, delayed fat necrosis, infection, cosmetic change, anxiety related to retained ablated tissue, quality of life, and cost is inconsistent. Publication bias remains possible, and negative or technically unsuccessful experiences may be underrepresented. Future studies should use prospective registration, consecutive enrollment, central imaging and pathology review, standardized ablation terminology, complete reporting of failures and rescue interventions, and prespecified patient-reported and economic endpoints (16, 19, 28).

Critical synthesis across studies suggests that inconsistent outcomes cannot be attributed solely to procedural variability. Other factors may contribute, including tumor biology, lesion location, breast composition, imaging visibility, device characteristics, post-ablation assessment strategy, and patient selection. For example, complete ablation rates may appear higher in cohorts restricted to small, ultrasound-visible lesions with clearly defined margins, whereas residual disease may be more frequent when tumors have extensive intraductal components or poorly delineated borders. Similarly, imaging-based follow-up may underestimate microscopic residual disease compared with immediate surgical excision, whereas ablate-and-resect designs may not fully capture real-world non-surgical pathways.

From a broader perspective, breast RFA sits within an expanding field of non-surgical local treatment. Advances in fusion imaging, three-dimensional ultrasound, contrast-enhanced ultrasound, MRI guidance, automated image registration, and computational treatment planning may improve target localization and margin assessment (24, 25, 28). The most useful innovations will be those prospectively linked to pathological completeness or long-term local control rather than to visually attractive procedural surrogates alone. A consensus core outcome set and interoperable procedural registry would allow technical parameters, imaging features, tumor biology, complications, rescue interventions, and oncologic outcomes to be compared across devices and institutions.

In parallel, the immunologic consequences of RFA—ranging from local microenvironment remodeling to potential systemic effects—have attracted growing attention. Preclinical data suggest that RFA can synergize with immunomodulatory strategies, including the use of immunoadjuvants in syngeneic breast tumor models (30). At the same time, mechanistic and translational studies indicate that incomplete ablation may induce a pro-tumorigenic inflammatory milieu and compromise antitumor immunity, emphasizing that any immunologic strategy must be grounded in reliable local control (31, 32).

Finally, exploratory applications in non-standard settings, including locally advanced disease, have been reported primarily as feasibility-oriented interventions (33). Although such experiences are not directly generalizable to contemporary early breast cancer practice, they underscore the importance of strict indication setting and safety-oriented system design when translating RFA into routine clinical use. When viewed across the broader thermal ablation literature, meta-analytic evidence supports the potential role of ablation-based strategies as an alternative local approach in highly selected small breast cancers, while also highlighting the need for standardized assessment and long-term outcome validation (29).

## Conclusion

Radiofrequency ablation can achieve high technical success and favorable 5-year local control in carefully selected patients with small, unifocal, image-visible early breast cancers. The 2026 RAFAELO results represent an important advance, but they support a protocolized multidisciplinary pathway—not unrestricted replacement of breast-conserving surgery. In practical terms, safe implementation requires three-dimensional tumor-centered electrode placement, tissue- and device-aware energy delivery, continuous thermal protection, objective recording of impedance, temperature and microbubble coverage, prespecified post-ablation imaging and biopsy criteria, and immediate access to definitive rescue treatment when completeness is uncertain. The next research priority is prospective comparative validation using standardized definitions, central imaging and pathology review, transparent reporting of failures and rescue interventions, and long-term assessment of local control, disease-free survival, cosmetic outcomes, quality of life, and cost-effectiveness. Until these data mature, breast RFA should be offered only within appropriately trained, audited, and multidisciplinary systems with explicit patient counseling regarding its benefits, uncertainties, and potential need for additional treatment.
